# Efficacy of night-time compression for breast cancer related lymphedema (LYNC): protocol for a multi-centre, randomized controlled efficacy trial

**DOI:** 10.1186/s12885-016-2648-8

**Published:** 2016-08-04

**Authors:** Margaret L. McNeely, Kristin L. Campbell, Marc Webster, Urve Kuusk, Karen Tracey, John Mackey

**Affiliations:** 1 Department of Physical Therapy, University of Alberta & Cross Cancer Institute, 2-50 Corbett Hall, Edmonton, Alberta T6G 2G4 Canada; 2 Department of Physical Therapy, University of British Columbia, Vancouver, British Columbia Canada; 3 Medical Oncology, Tom Baker Cancer Centre, Alberta Health Services & Department of Oncology, University of Calgary, Calgary, Alberta Canada; 4 Department of Surgery, Faculty of Medicine, University of British Columbia & British Columbia Cancer Agency, Vancouver, British Columbia Canada; 5 Clinical Trials Unit, Cross Cancer Institute, Alberta Health Services, Edmonton, Alberta Canada; 6 Medical Oncology, Cross Cancer Institute, Alberta Health Services & Department of Oncology, University of Alberta, Edmonton, Alberta Canada

**Keywords:** Breast cancer, Lymphedema, Compression therapy, Physical therapy

## Abstract

**Background:**

Lymphedema is a prevalent long-term effect of breast cancer treatment that is associated with reduced quality of life. More recent observational data suggest that the addition of night-time compression to day-time use of a compression garment results in better long-term control of arm lymphedema. The primary objectives of the randomized controlled phase of the trial are to determine the efficacy of night-time compression on arm lymphedema volume maintenance and quality of life in breast cancer survivors who have completed intensive reduction treatment for their lymphedema.

**Methods/Design:**

The study will be a parallel 3-arm, multi-centre randomized fast-track trial. A total of 120 women with breast cancer related lymphedema will be recruited from 3 centres in Canada and randomized to group 1: Day-time compression garment alone or Group 2: Day-time compression garment + night-time compression bandaging or Group 3: Day-time compression garment + use of a night-time compression system garment. The duration of the primary intervention period will be 12 weeks. The follow-up period after the intervention (weeks 13 to 24) will follow a longitudinal observational design. The primary outcome variables: differences from baseline to week 12 in arm volume and quality of life (Lymphoedema Functioning, Disability and Health Questionnaire: Lymph-ICF). Secondary outcomes include bioimpedance analysis, sleep disturbance and self-efficacy. All measurements are standardized and will be performed prior to randomization, and at weeks 6, 12, 18 and 24.

**Discussion:**

The use of night-time compression as a self-management strategy for chronic breast cancer related lymphedema is seen as an innovative approach to improve long-term control over the condition. This trial aims to advance the knowledge on self-management strategies for lymphedema.

**Trial registration:**

This trial was registered at clinicaltrials.gov on July 9^th^, 2014 (NCT02187289)

## Background

Breast cancer is the most common cause of cancer in Canadian women with an estimated 25,000 developing the disease in 2015 [1]. With a current 5-year survival rate of 88 %, a substantial number of women in Canada are living with a history of breast cancer [1]. One of the more frequent complications following treatment for breast cancer is lymphedema, a significant swelling of the arm that occurs on the surgical side. Lymphedema is a lifelong condition that tends to worsen over time [2]. Recent data suggest that approximately 21 % of women who undergo treatment for their breast cancer are diagnosed with lymphedema [3]. Of these cases, approximately half will develop chronic progressive lymphedema [4, 5].

The impact of lymphedema on the breast cancer survivor is often profound, resulting in negative changes in self-image, increased anxiety, and poorer quality of life [6, 7]. Overtime, lymphedema can create considerable disability with recurrent infections in the limb, functional impairment, and pain [2, 8]. Many survivors see lymphedema as an ongoing reminder of cancer to themselves, friends, and family [6]. Compounding the problem is that survivors report receiving limited support and inadequate or conflicting information on the condition and its treatment from healthcare providers [6]. In a matched cohort analysis of breast cancer survivors with and without lymphedema, those with lymphedema had significantly higher medical costs at 2 years (range of $14,877 to $23,167) and were twice as likely to develop an infection in the limb as compared to survivors without lymphedema [8].

At present, there are no known curative treatments, either surgical or pharmacological, for lymphedema [9]. Rehabilitative treatments, also called conservative treatments, are prescribed to reduce and maintain limb size, to restore function, to reduce pain, and to improve the appearance of the limb [9, 10]. An initial intensive treatment program is usually prescribed to reduce the lymphedema (Phase I) and is followed by a maintenance phase (Phase II) which aims to promote life-long maintenance [11]. Decongestive Lymphatic Therapy (DLT) is a 2-to-4 week intensive treatment program comprised of daily manual lymph drainage (MLD) massage (a specialized massage technique), wrapping with multi-layered short-stretch compression bandages (worn 23 h per day), exercise, and education [12]. The current recommended intensive treatment approach for Phase I is DLT, with or without the inclusion of the MLD massage component [9]. Following treatment to reduce the swelling, a compression sleeve is prescribed. Compression sleeves represent current standard of care in lymphedema maintenance (Phase II) [9].

A prospective cohort study including 682 women with breast cancer related lymphedema examined the ability of women to maintain arm lymphedema volume following an intensive course of reduction treatment for lymphedema [13]. In the study, the authors observed a gradual increase in lymphedema volume over time among study participants, with the greatest increases seen in the first year. The risk of treatment failure, defined as a >50 % relapse in arm lymphedema volume from baseline levels, was 38.1 %, 53.1 %, and 64.8 % at 1, 2, and 4 years respectively [13]. Interestingly, women who wore a day-time compression sleeve and also wrapped their affected limbs with compression bandages (CB) at night (a minimum of 4 nights per week), had a significantly reduced risk of relapse in arm lymphedema volume [hazard ratio, 0.53, (0.34–0.82), *P* = 0.004] [13]. The authors concluded that successful control of lymphedema may be dependent on the combined use of the compression sleeve and CB during day and night periods, respectively.

**Fig. 1:**
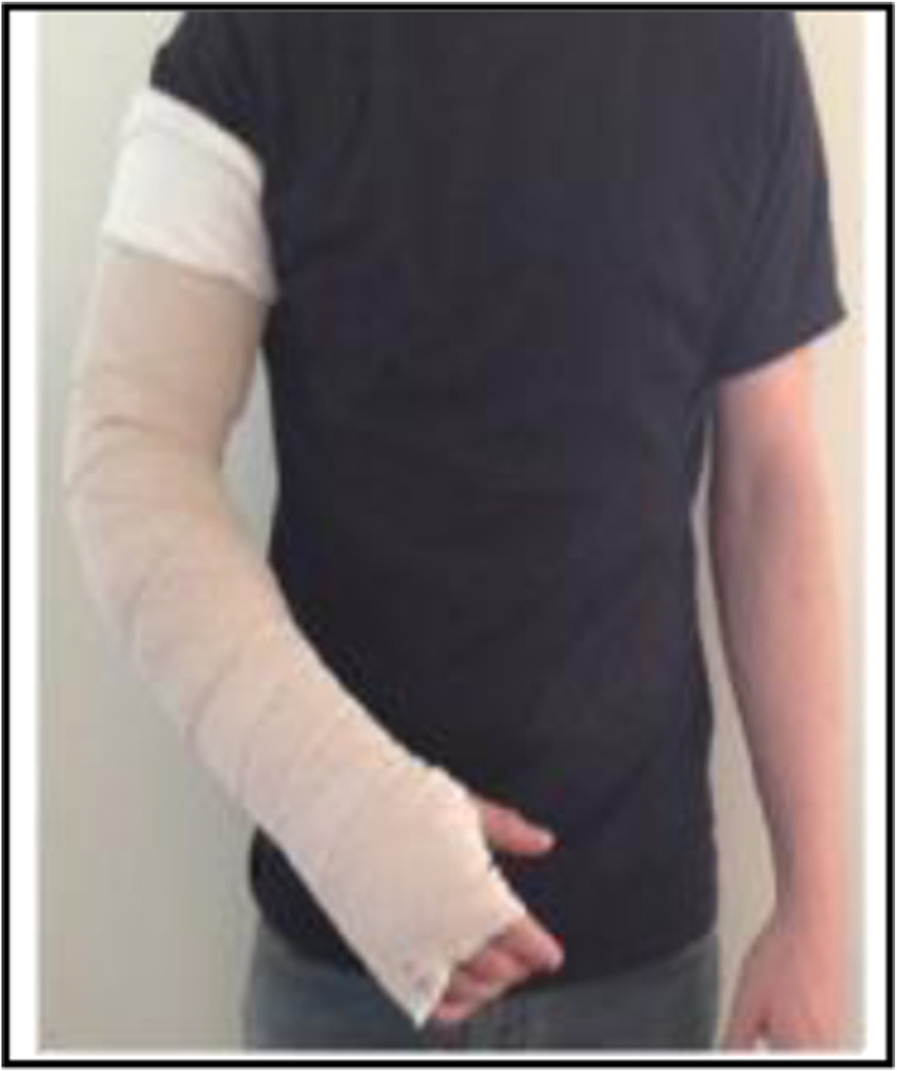
Compression bandaging

**Fig. 2:**
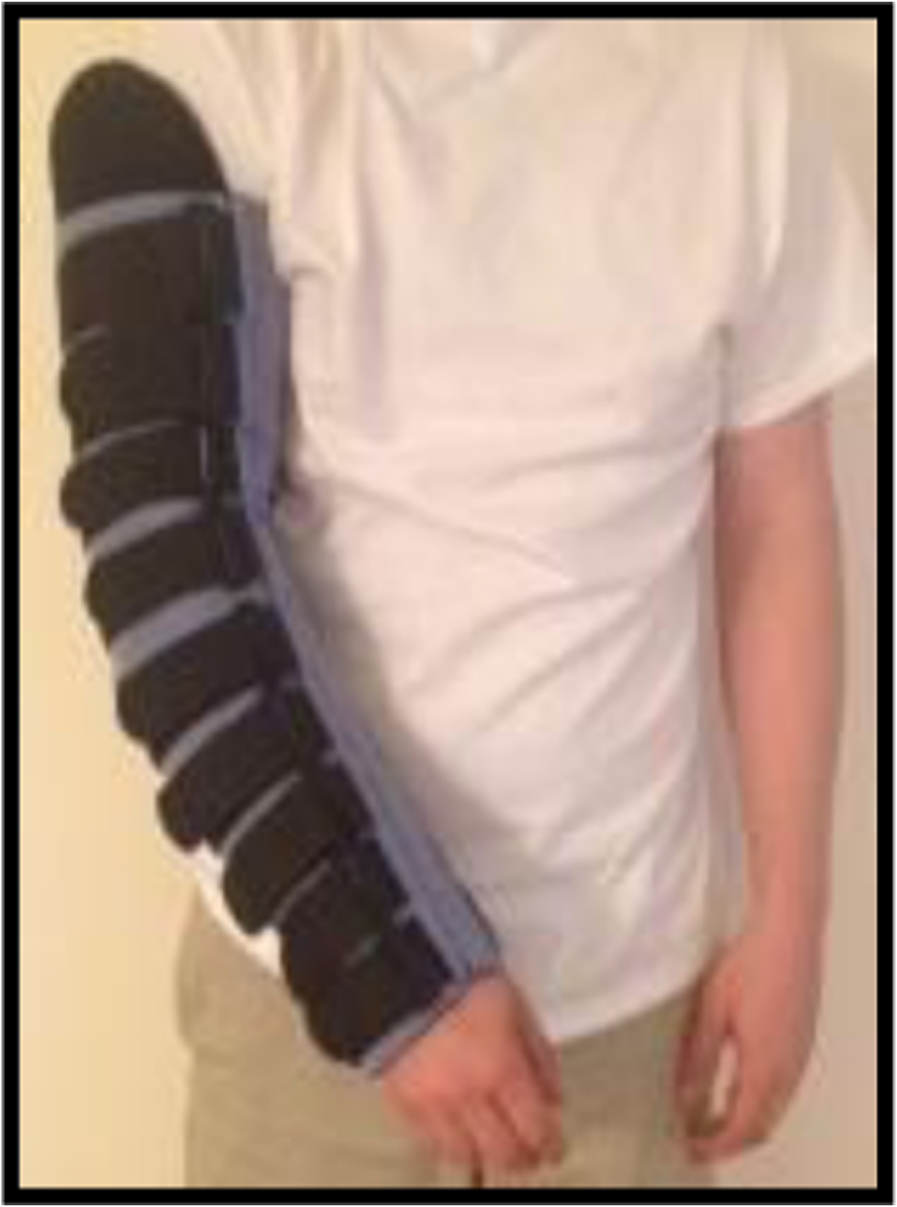
Example of strapped version of a night-time compression system garment

Applying compression at night is an option that is usually presented to the survivor when the condition is advanced or when relapses in symptoms and/or arm lymphedema volume occur [9]. When night-time compression is indicated, the survivor is taught how to apply the bandages to the arm [Fig. 1]. Although many survivors are able to bandage their arm independently, a family member or significant other is often taught the bandaging technique and assists the survivor at night. Various types of night-time compression system garments (NCSGs) have been designed as alternatives to CB. NCSGs are simple to use, quick to apply to the limb, and can be easily adjusted to provide the appropriate amount of pressure. In general, these compression system garments apply gentle gradient pressure to the limb through a garment with a foam liner and a series of adjustable straps [Fig. 2]. The garments are non-elastic and provide low resting pressure on the limb, making them safe to wear while sleeping at night. NCSGs provide an alternative self-management strategy for women to reduce the time burden associated with compression bandaging as well as for those unable to appropriately apply CB to their arm (e.g., older individuals with mobility issues and/or those who live alone). Although NCSGs are already available for purchase, as with many marketed treatment options for lymphedema, little is known about their effectiveness.

### Hypotheses of randomized controlled trial (Phase I)

1. Standard care plus the addition of CB will provide statistically significant improved management of **arm lymphedema volume** compared to standard care alone at 12-weeks.
2. Standard care plus the addition of NCSG will provide statistically significant improved management of **arm lymphedema volume** compared to standard care alone at 12-weeks.
3. Standard care plus the addition of NCSG will provide statistically significant benefit in **quality of life**, **sleep and self**-**efficacy** over standard care plus CB and standard care alone at 12-weeks.

### Objectives

The primary objectives of the randomized controlled phase of the trial are to determine the effect of night-time compression on arm lymphedema volume maintenance and quality of life in breast cancer survivors who have completed intensive reduction treatment for their lymphedema. These questions are seen as valuable for informing implementation of night-time compression as a self-management strategy for lymphedema in the clinical setting. The primary objectives of follow-up are to evaluate the long-term adherence to use of night-time compression and the timing of the intervention effect. As well, this follow-up period will allow us to better understand who may benefit the most (or least) from the addition of night-time compression.

## Methods/Design

**Fig. 3:**
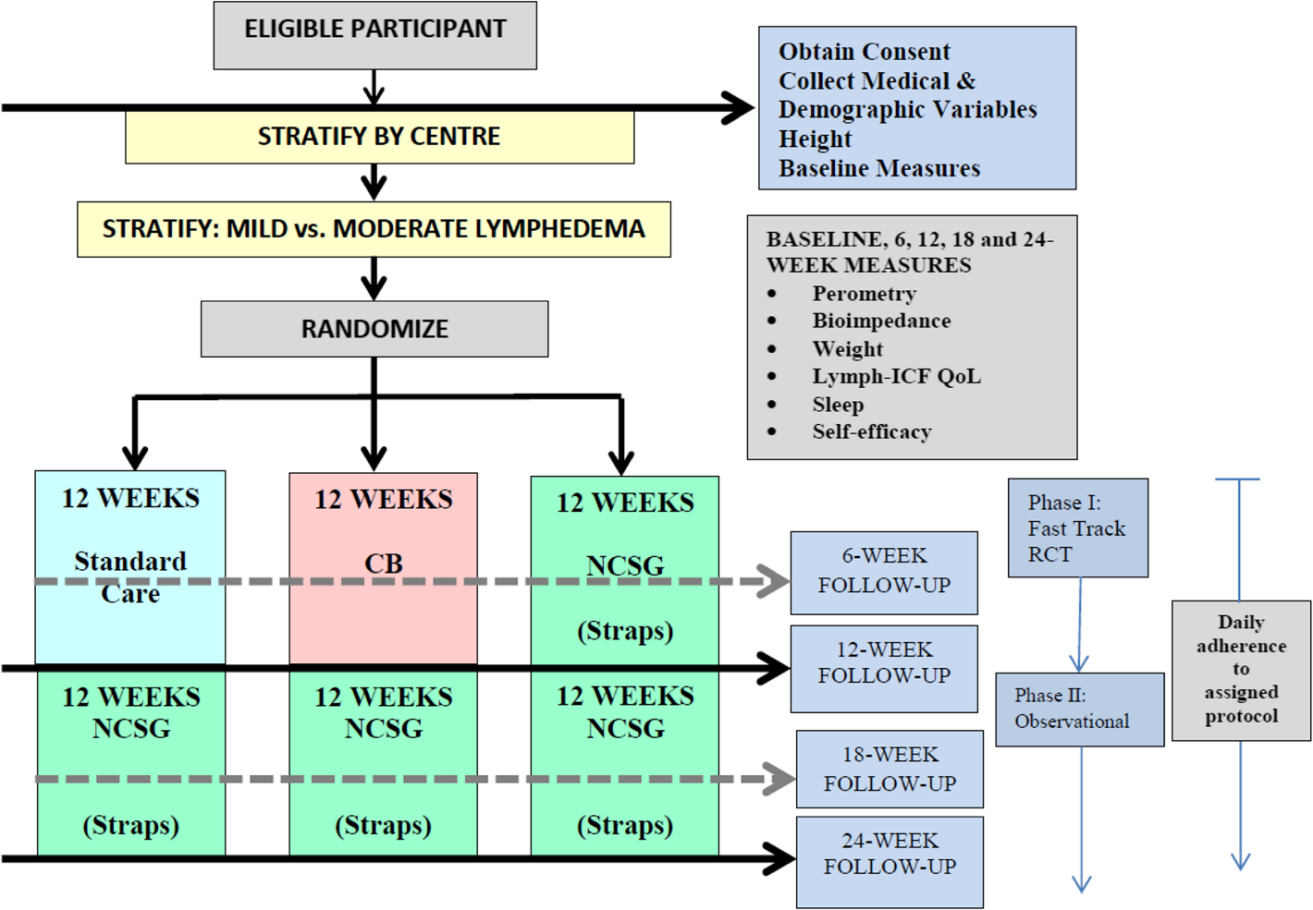
Study schema

The study will be a parallel 3-arm, multi-centre randomized fast-track trial including the Cross Cancer Institute (CCI) in Edmonton, Tom Baker Cancer Centre (TBCC) in Calgary and Mount St. Joseph’s Hospital (MSJ) in Vancouver. Women enrolled in the study will be stratified by accruing centre (CCI, TBCC, MSJ) and by lymphedema severity (i.e., mild versus moderate lymphedema as per the classification criteria of International Society of Lymphology) [9, 14], and then randomly assigned to one of three groups: (1) standard care (day-time use of a compression sleeve alone); (2) standard care plus night-time compression by self-administered or assisted CB; (3) standard care plus night-time compression by a traditional NCSG. The duration of the primary intervention period will be 12 weeks. Results from the pilot study and previous case studies suggest a small initial reduction in arm lymphedema volume from administration of the traditional strapped NCSG over the first 6 weeks with stabilization of arm volume (plateau) occurring thereafter. Thus, the 12-week intervention period is deemed sufficient to observe an intervention effect and was chosen to align with current clinical follow-up time periods. Following the 12-week intervention period, participants in the CB and standard care groups will be measured for a NCSG. Participants in these two groups will follow the protocol as per the NCSG group during weeks 13 to 24 [Fig. 3].

The randomized fast-track trial (with delayed assignment to NCSG for both comparison groups) is seen as a more acceptable design to patients, families and staff, given that the majority of eligible participants will have a strong preference for assignment to the NCSG group. Moreover, we feel this design, which was used in the pilot study, will optimize recruitment and retention of the comparison group participants in the trial.

The follow-up period after the intervention (weeks 13 to 24) will follow a longitudinal observational design and provides an opportunity to examine factors related long-term adherence to night-time compression.

### Participants

The following eligibility criteria will be used to determine participant inclusion in the trial:

1. Women with a histological diagnosis of breast cancer experiencing lymphedema in the ipsilateral arm such that there is a minimum 200 ml or 10 %, and maximum 40 % increase in arm volume over the unaffected arm (indicative of mild to moderate lymphedema) [14].
2. Survivors must have completed all primary and adjuvant cancer treatments (with the exception of hormonal treatment) by a minimum of 1 month prior to randomization.
3. Survivors must be in or entering the lymphedema maintenance phase and agreeable to **not** pursuing any other lymphedema maintenance treatments beyond day-time use of a compression sleeve.
4. Survivors must have their own properly fitted compression sleeve for day-time maintenance and must agree to wear their day-time sleeve as per standard care for a minimum of 12 h per day.
5. No current use of night-time compression for maintenance. Any survivor who has trialed a NCSG/performed night-time CB in the past year for maintenance purposes must observe a one-month washout period before entering the trial.

### Survivors will be excluded if they present with any of the following

1. Clinical or radiological evidence of active breast cancer, either local or metastatic.
2. History and clinical diagnosis of bilateral arm lymphedema.
3. Serious non-malignant disease, such as renal or cardiac failure, which would preclude daily treatment and follow-up.
4. Survivors for whom compression is contraindicated, such as those with untreated infections, skin irritation/rash or thromboses in the affected arm.
5. Psychiatric or addictive disorders, as determined by the referring physician, which preclude obtaining informed consent or adherence to the protocol.
6. Unable to comply with the protocol, measurement, and follow-up schedule due to factors, such as vacation during the study period.

### Interventions

Management in both the experimental and comparison groups will follow recommendations of the Canadian Breast Cancer Initiative’s Steering Committee for Clinical Practice Guidelines for the Care and Treatment of Breast Cancer [15]. Survivors will be provided with advice concerning good skin care, regular exercise, maintenance of a healthy body weight, and the use of a day-time compression sleeve.

#### Group 1: standard care: day-time use of compression sleeve alone

Women randomized to the standard care group will receive standard care for lymphedema maintenance. In this group of the trial, each participant will be instructed to wear their day-time compression sleeve with or without a gauntlet/glove (worn if swelling in the hand and fingers), providing a minimum of 30 mm Hg of pressure, for 12 h per day, each day of the week. A 6-week follow-up will be performed to evaluate response and adherence to the standard care protocol. At week 12, women in this arm of the trial will be fitted for a NCSG and will follow the protocol outlined in the experimental arm of the trial.

#### Group 2: standard care plus night-time multi-layered Compression Bandaging (CB group)

Women randomized to the night-time CB group will be instructed in the application of night-time multi-layered CB by the physical therapist. Women who have performed nighttime multi-layered CB as part of their past maintenance program will attend a review session to ensure appropriate bandaging technique and application. Educational materials related to compression bandaging application will be provided. Participants will have a 2-week phase in period, following which they will be asked to wear the CB at night while sleeping for a minimum of 8 h per night, a minimum of five nights per week for 4 weeks (weeks 3–6). As per standard of care, each participant will be required to wear their day-time compression sleeve with or without a gauntlet/glove, providing a minimum of 30 mm Hg of pressure, for 12 h per day, each day of the week. A 6-week follow-up will be performed to evaluate response and adherence to the CB protocol. At that point, maintenance CB a minimum of 3 times per week will be introduced. At week 12, women in this arm of the trial will be fitted for a NCSG and will follow the protocol outlined in the experimental arm of the trial.

#### Group 3: standard care plus Night-Time Compression System Garment (NCSG group)

Women randomized to the immediate NCSG group will be measured for a NCSG. Once the garment has arrived, participants in this group will be instructed in use of their NCSG by the physical therapist. Educational materials related to garment application and maintenance will be provided. Participants will have a 2-week phase in period, following which they will be asked to wear the NCSG at night while sleeping for a minimum of 8 h per night, a minimum of five nights per week for 4 weeks (weeks 3–6). As per standard of care, each participant will be required to wear their day-time compression sleeve with or without a glove/gauntlet, providing a minimum of 30 mm Hg of pressure, for 12 h per day, each day of the week. A 6-week follow-up will be performed to evaluate response, adherence to the protocol and to address any issues with wear of the NCSG. At that point, maintenance NCSG a minimum of 3 nights per week will be introduced. After the 12-week assessment, participants in this arm of the trial will have the option to continue use of their NCSG.

### Recruitment

Ethics approval and signed informed consent of participants is required. Recruitment started after approval was received from all respective Health Research Ethics Committees (Health Research Ethics Board of Alberta: Cancer Committee and the University of British Columbia Ethics Board). The estimated timeline for recruitment is 18–24 months. Potential participants will be identified and screened for eligibility by the respective research coordinators through the Rehabilitation Medicine Departments at the CCI in Edmonton, the TBCC in Calgary, and MSJ in Vancouver. Initial eligibility will be determined by chart review and, if necessary, verified through contact with the participant’s physician. Interested participants will be scheduled for a baseline visit to obtain written consent and for final determination of eligibility. It is anticipated that 10 to 15 breast cancer survivors with lymphedema (per month) will be eligible among the three centres and that 5–8 women (per month) will agree to participate. Follow-up measurements will occur at week 6, 12 (end of RCT portion), 18, and 24 following the start of the assigned intervention for the participant. The last participant entering the trial will therefore undergo her 24-week follow-up measurement approximately 30 months after recruitment begins.

### Baseline data and endpoints

During the baseline visit, demographic and clinical information will be collected on participants. Medical data will be abstracted from the medical health records.

### Primary endpoints

*Arm volume* is a commonly used metric to evaluate lymphedema severity and to inform decision-making in the clinical setting. Lymphedema will be objectively measured using the Perometer (Pero-systems, Wipputal, Germany). The Perometer is an optoelectric limb volumeter that uses infrared technology to quantify limb volume and determine inter-limb difference. Measurement with the perometer takes approximately 3 min to complete. The perometer is a valid, reliable, and sensitive method for quantifying limb volume [16–18].

*Quality of life* will be measured using the condition-specific Lymphoedema Functioning, Disability and Health Questionnaire (Lymph-ICF) [19]. This 29-item questionnaire was developed to assess quality of life specific to lymphedema and to monitor progress of treatment on function (i.e., movement, activities) and symptoms related to lymphedema (i.e., pain, discomfort, heaviness, and tension) as well as to assess activity limitations and participation restrictions. The Lymph-ICF has been tested and shown to be valid and reliable [19]. As well, general health-related quality of life will be assessed at each visit by using the RAND 36-item Short Form Health Survey (SF-36). These two questionnaires will take approximately 10–15 min to complete.

### Secondary endpoints

Secondary endpoints will include bioimpedance analysis, adherence, sleep disturbance, and self-efficacy.

*Bioimpedance analysis* (BIA) will be used to assess *extracellular fluid status* within the arm. BIA measures the impedance of flow from a low alternating electrical current that is applied to the body through a skin electrode. BIA records impedance values for each limb and provides an index that correlates with quantitative measurements of volume increase in limb size seen in the arm with lymphedema [20]. BIA is a simple, painless procedure that takes less than 5 min to perform. BIA has been shown to be a more sensitive measure than arm volume in detecting changes in lymphedema in those with **early mild lymphedema** but less reliable in those with long-standing lymphedema [20]. This measurement will provide information on extracellular fluid changes that complements the data on arm lymphedema volume.

*Sleep disturbance* will be assessed using the RAND Medical Outcomes Survey Sleep Survey [21]. The survey measures 6 key dimensions of sleep: sleep initiation, maintenance, quantity, adequacy, drowsiness, and respiratory impairments (e.g. shortness of breath, snoring). The sleep scale will be used to assess the impact, if any, of wearing night-time compression on sleep quality. The questionnaire takes 5 min to complete.

#### Self-efficacy of lymphedema management

Self-efficacy will be assessed based on the 6-item Chronic Disease Self-Management scale [22] modified for use with lymphedema. The scale assesses several domains that are common across chronic diseases such as symptom control, role function, emotional functioning, and communicating with physicians. The questionnaire takes 3-to-5 min to complete.

*Body height* will be recorded at baseline and *body weight* at baseline and each follow-up time point.

#### Adherence

Participants will be asked to record their adherence (i.e. days per week, hours per day) to their assigned compression therapy treatment in a daily journal. Treatment-specific outcomes such as the time required for administration of night-time compression, reasons for removal of the garment at night and independence/assistance in application will be included in the diary. Participants will be asked to return the completed diary for each respective 6-week time period at the subsequent follow-up visit. We will also record any additional appointments required to teach CB or to assess wear/fit of the NCSG.

### Adverse events

*Adverse events* related to the application of compression (i.e. skin breakdown or reaction, such as rash) will be recorded. Any *adverse events* related to the condition of lymphedema (i.e. infection/cellulitis or blood clot in the arm) will be verified with the participant’s physician. If this occurs, as per standard care, treatment with compression will be stopped until deemed safe to resume by the physician. We will also record any relapses in arm lymphedema volume (>50 %) that require more intensive reduction treatment.

### Allocation concealment and method of randomization

Participants will be randomized in a 1:1:1 ratio to Standard Care, CB or NCSG using a secure central randomization service administered by the Clinical Trials Unit of the Cross Cancer Institute.

### Blinding

At each measurement point starting after the baseline assessment through the RCT portion of the trial (i.e., 6 week and 12 week follow-ups), an Independent Assessor unaware of treatment allocation (blinded) will perform the measurements of arm volume using the perometer, bioimpedance analysis, and body weight. The Independent Assessor will also administer the outcome measures for sleep disturbance, self-efficacy, and quality of life. The Adherence Diary will be collected by the Research Coordinator at each follow-up visit. Follow-up sessions will take place in the respective rehabilitation department and each session will take 30 to 45 min to complete. Blinding of participants and practitioners will not be possible as participants are aware of the compression therapy treatment they are receiving. Study garments will be purchased at cost from participating industry partners through the grant funds. In the event that a study participant is unable to continue with their assigned treatment, they will remain in their randomized group to preserve the intention-to-treat principle. Study participants will be free to withdraw at any time but will be invited to continue to attend for scheduled follow-ups for the duration of the study.

### Sample size for randomized fast-track trial phase

The sample size required per group to detect at least a minimal clinically important mean difference of 20 % (SD ±25) in arm lymphedema volume in favor of either the standard care + NCSG, standard care + CB group and standard care alone is 36 participants. A total sample of 108 participants will achieve a power of 86 % with a significance level of 0.05 using a one way ANOVA test. The sample size required per group to detect at least a minimal clinically important difference on the Lymph-ICF quality of life scale of 15 points out of 100 (SD ±22) between the standard care + CB group and the standard care + NCSG group is 36 participants. A total sample of 108 will achieve a power of 80 % with a significance level of 0.05 using a two-sided Mann-Whitney test assuming the actual distribution is normal. To allow for an estimated 10 % loss to follow-up and non-compliance/cross-over of the standard care group, we will recruit an additional 12 patients for a total of 120 participants (40 participants in each of the three treatment groups).

### Statistical analyses

Baseline medical and demographic characteristics, arm dominance relative to the lymphadematous arm, and adverse events of the three groups will be compared using one-way ANOVA for continuous data and Pearson’s Chi-square tests for categorical data. The primary analysis will compare the groups with regard to percent excess lymphedema volume and quality of life at 12-weeks using a one-way ANOVA. General linear models will be used to adjust for centre and lymphedema category (mild or moderate), and to evaluate the treatment effect in subgroups defined by the strata. Sleep quality, self-efficacy, and adherence-related outcomes will be analyzed by repeated measures modeling and one-way ANOVA on change scores.

Descriptive data on accrual rates will include the number of women screened for eligibility, the number eligible, the number agreeing to participate, and reasons for refusal to participate. Data on adherence will be collected from all women throughout the 24-week study period. Adherence data will include days worn per week and hours worn per 24-h period, as appropriate, for each of the following: 1) NCSG; 2) CB; 3) day-time compression sleeve. We will also examine adherence during weeks 13 to 24 to the newly assigned NCSG for the standard care and standard care + CB groups, and ongoing adherence during weeks 13 to 24 in the originally assigned standard care + NCSG group. Analyses of primary outcomes will be performed at the end of the RCT portion of the trial. Within group analyses will also be conducted for primary and secondary outcomes from weeks 13 to 24 following completion of all follow-up measures.

### Trial management

A primary Trial Coordinator will be assigned by the CTU for the Edmonton site and this individual will oversee the day-to-day operations of the project and report directly to Dr. McNeely. The primary Trial Coordinator revised the standard operating procedures used in the pilot study in consultation and in collaboration with the research team members. The Calgary and Vancouver sites have Research Coordinators/Physical Therapist (PT) who work under the supervision of Drs. McNeely and Campbell, respectively. The respective Research Coordinator-PT will be responsible for booking of participant appointments, teaching of self-bandaging to participants, measurement and fitting of participants for night-time compression system garments, and provide assistance and oversight for data collection and data entry. Case report form development, randomization, data entry, quality assurance and data analysis will be carried out by the CTU. To protect the identity of study participants, forms will be labeled with the participant study identification number and initials only. Original case report forms will be sent to the CTU. Copies of the original forms will be secured in the respective departments. The CTU will provide biostatistical support for the statistical analyses of data.

## Discussion

### Integrated knowledge translation

A survivor-representative, physical and occupational therapists, rehabilitation department administrators, and industry were asked to provide input on knowledge translation goals and methods. Consumer interests include information on the value of the garment for lymphedema, garment choice and options and prescription variables (i.e. how long to wear, how many nights per week). Goals of the healthcare practitioners include understanding the relative benefit of NCSGs and resolving when to best prescribe garments. Other questions revolve around independence in application and time benefit for the survivor. Industry interests include questions on NCSG comfort and effectiveness.

Knowledge translation methods will include webinars, workshops and small group face-to-face sessions for survivor groups in Alberta and British Columbia as well as front-line health care practitioners working directly with breast cancer survivors in these two provinces. Connections with Alberta’s Community Cancer Support Network, Alberta CancerControl Supportive Care Council, British Columbia Cancer Agency, the Alberta Lymphedema Association and the British Columbia Lymphedema Association will ensure that information gained from the study will be delivered across the regions (i.e, newsletter and email communications). Nationally, research findings will be disseminated to the Canadian Physiotherapy Association Oncology Division (i.e. study briefing in newsletter), and the Canadian Lymphedema Framework (i.e., lay language summary in lymphedema magazine Pathways).

### Limitations

Limitations of the current design include the requirement that participants be able and willing to be randomized to one of the three groups for a 12-week period. For this reason, we anticipate women who regularly administer CB at night and/or attend regular maintenance sessions of MLD will not be interested, or eligible to take part in the trial.

### Progress to date

As of September 2015, 39 women with breast cancer related lymphedema have been randomized and no adverse events have occurred.

### Relevance and innovation

As the numbers of survivors of breast cancer continues to grow, there is need for supportive care interventions to address the late and long-term effects of cancer treatment. Lymphedema is a prevalent long-term effect of treatment that is associated with reduced quality of life. There are currently a number of products and devices on the market purported to reduce and/or help manage lymphedema, many of which can be purchased directly by the survivor on the internet. However, there is a paucity of quality research to support the use of many of these products and interventions for lymphedema, including NCSGs. For these reasons, healthcare practitioners are unsure of how to best advise survivors. This study will prospectively examine the effect of the addition of night-time compression to current standard care on the quality of life of breast cancer survivors with lymphedema. While these treatment techniques have been used clinically for many years, the evolution of night-time compression therapy as a self-management strategy is the innovative aspect of this approach and research. In this way, this study will help advance the knowledge on self-management strategies for lymphedema.

## Abbreviations

CB: Compression bandaging
DLT: Decongestive lymphatic therapy
MLD: Manual lymphatic drainage
NCSG: Night-time compression system garment
